# A Mechanistic Perspective on PEX1 and PEX6, Two AAA+ Proteins of the Peroxisomal Protein Import Machinery

**DOI:** 10.3390/ijms20215246

**Published:** 2019-10-23

**Authors:** Ana G. Pedrosa, Tânia Francisco, Maria J. Ferreira, Tony A. Rodrigues, Aurora Barros-Barbosa, Jorge E. Azevedo

**Affiliations:** Instituto de Investigação e Inovação em Saúde (i3S), Instituto de Biologia Molecular e Celular (IBMC), Instituto de Ciências Biomédicas de Abel Salazar (ICBAS)—Universidade do Porto, Rua Alfredo Allen, 208, 4200-135 Porto, Portugal; ana.pedrosa@i3s.up.pt (A.G.P.); taniaf@ibmc.up.pt (T.F.); maria.ferreira@i3s.up.pt (M.J.F.); tonyr@ibmc.up.pt (T.A.R.); aurora.barbosa@i3s.up.pt (A.B.-B.)

**Keywords:** AAA+ ATPase, peroxisome, PEX1, PEX6, PEX5, protein translocation

## Abstract

In contrast to many protein translocases that use ATP or GTP hydrolysis as the driving force to transport proteins across biological membranes, the peroxisomal matrix protein import machinery relies on a regulated self-assembly mechanism for this purpose and uses ATP hydrolysis only to reset its components. The ATP-dependent protein complex in charge of resetting this machinery—the Receptor Export Module (REM)—comprises two members of the “ATPases Associated with diverse cellular Activities” (AAA+) family, PEX1 and PEX6, and a membrane protein that anchors the ATPases to the organelle membrane. In recent years, a large amount of data on the structure/function of the REM complex has become available. Here, we discuss the main findings and their mechanistic implications.

## 1. Introduction

Many newly synthesized proteins have to be translocated across at least one biological membrane in order to reach their final destination. This is the case for thousands of eukaryotic proteins that are synthesized on cytosolic ribosomes but belong to organelles such as mitochondria, the endoplasmic reticulum, and peroxisomes, and for bacterial proteins that are destined to the periplasm or outer membrane [1,2,3,4,5,6]. Translocation of these proteins across (a) membrane(s) is a highly specific process relying on (1) the presence of targeting sequences in their primary structures, (2) receptors that specifically recognize these targeting signals, and (3) protein modules that form the membrane channels/pores through which translocation occurs [7]. In addition, some type of energy is used to provide directionality to the protein translocation step. Frequently, energy from ATP or GTP hydrolysis is used to trigger conformational alterations in components of the translocation machinery which in turn either push the protein substrate through the membrane (power stroke mechanisms) or bind and retain the substrate at the trans side of that membrane (molecular ratchet mechanisms) [8,9,10]. In at least one case—protein import into mitochondria—the electric component of the membrane potential is explored to induce electrophoretic movement of the translocation substrate [11]. In other systems, translocation directionality is attained through strong molecular interactions established between components of the translocation machinery which end up pushing the substrate across the membrane (regulated self-assembly mechanisms; e.g., peroxisomal matrix protein import, type V and VI bacterial secretion systems, and AB-type toxins; [3,5,12]). In some of the latter cases energy from ATP hydrolysis is still needed but only to reset the protein translocation machinery [13]. This is the case of the peroxisomal matrix protein import machinery, which undergoes structural remodeling after each protein translocation event in order to remain active. The proteins involved in this resetting process are long known but only recently did we start to understand how they work. Here we describe these proteins discussing the major findings gathered in recent years.

## 2. Going through the Peroxisomal Membrane—the Docking/Translocation Module and PEX5

**Figure 1:**
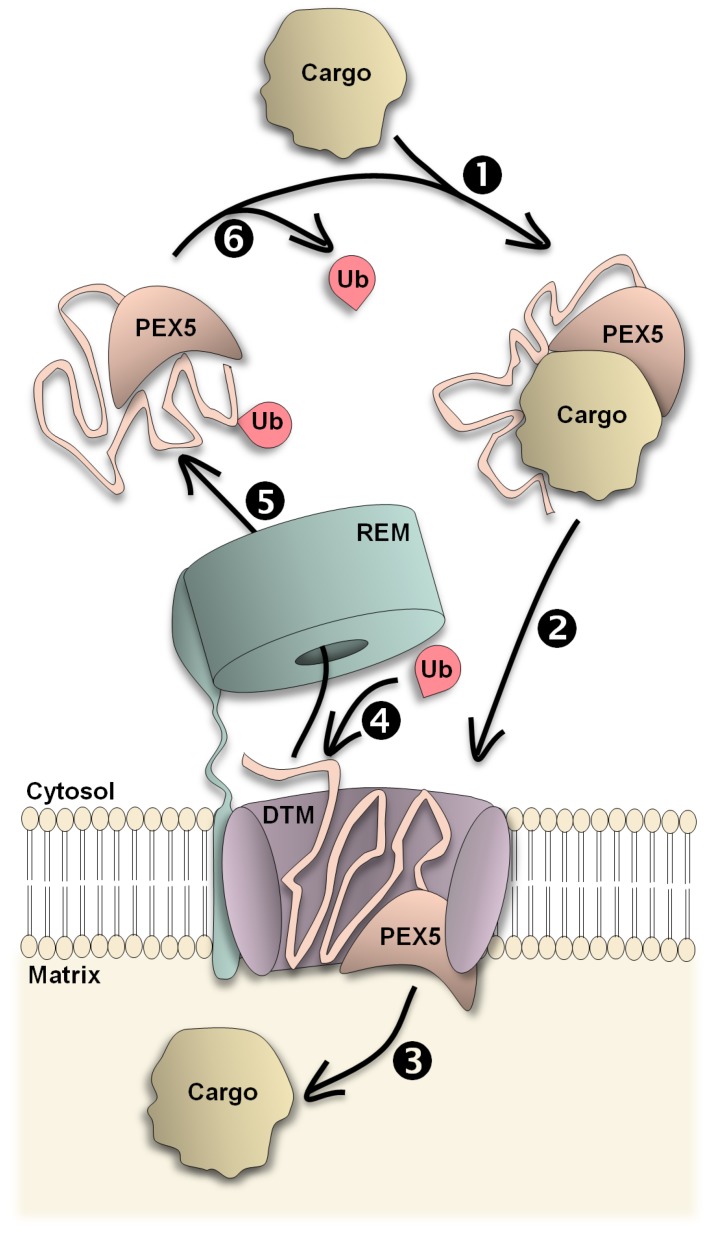
Import of peroxisomal matrix proteins. A newly synthesized matrix protein (“Cargo”) is recognized by PEX5 in the cytosol (step 1). The PEX5-cargo complex interacts with a multisubunit protein complex at the peroxisome membrane, the Docking/Translocation Module (“DTM”; step 2), comprising PEX13, PEX14, and the RING peroxins PEX2, PEX10, and PEX12. Then, PEX5 becomes inserted into the DTM thus pushing the cargo protein across the membrane and into the organelle lumen (step 3). Afterwards, PEX5 is monoubiquitinated (Ub) at an N-terminally conserved cysteine residue (step 4) and is then extracted back to the cytosol by the action of the Receptor Export Module (REM; step 5), which comprises the AAA+ proteins PEX1 and PEX6 plus a membrane anchor (PEX26/PEX15/APEM9; see main text for details). In the cytosol, PEX5 is rapidly deubiquitinated (step 6) and a new round of import can be initiated. The irregular zigzag line represents the intrinsically disordered N-terminal half of PEX5, whereas the crescent-shaped form represents the globular PTS1-binding domain of PEX5.

Peroxisomal matrix proteins are synthesized on cytosolic ribosomes and rapidly transported to the organelle by a shuttling receptor [14,15]. The mechanism of this pathway can be described by drawing an analogy with a syringe (see Figure 1; [3]): the shuttling receptor (the “plunger”) binds a cargo protein in the cytosol, an event that triggers its insertion into a peroxisomal transmembrane complex (the “syringe barrel”) with the concomitant translocation of the cargo across the organelle membrane. No nucleoside triphosphate hydrolysis or membrane potential are needed for the translocation step—the driving force derives from the strong and multivalent interactions established between the “plunger” and the “barrel” [16,17,18]. After delivery of the cargo, the “syringe” is disassembled, i.e., the “plunger” is extracted from the “barrel” so that a new cycle of protein transportation can be started. This is the only part of the pathway where energy from ATP hydrolysis is required (reviewed in ref. [3]).

In mammals, plants and many other organisms the “plunger” is PEX5, a monomeric and largely intrinsically disordered protein that recognizes in an autonomous manner the vast majority of proteins destined to the peroxisomal lumen [19,20,21,22,23,24,25,26,27,28]. These proteins possess the Peroxisomal Targeting Signal (PTS) Type 1, a small peptide at their C-termini frequently ending with the sequence SKL [29]. A small number of proteins possess instead a PTS Type 2, a degenerated nona-peptide present at their N-termini [30]. PTS2 proteins are also delivered to the peroxisome matrix by PEX5 but in this case an auxiliary protein PEX7 is required to strengthen the PEX5–PTS2 cargo protein interaction [31,32,33,34,35,36,37,38,39,40]. The situation in yeasts and fungi is slightly different. These organisms also rely on PEX5 to transport PTS1 proteins to the peroxisome [19,22,28,41]. However, they use a different “plunger” to import PTS2 proteins, also in a PEX7-dependent manner, and may possess still another shuttling receptor to transport some PTS1 proteins to the organelle under certain growth conditions [42,43,44,45,46,47]. Importantly, all these additional yeast/fungi “plungers” display structural similarities to PEX5 and thus, they are believed to function in an identical manner [42,43,45,48,49].

The polypeptide composition of the “syringe barrel”, referred to as the Docking/Translocation Module (DTM), has been known for many years. It comprises five core components, all transmembrane proteins. These are PEX13 and PEX14, each probably present in multiple copies per DTM, and three proteins containing a really-interesting-new-gene finger domain (RING), PEX2, PEX10, and PEX12 [50,51]. The overall structure of the DTM remains unknown. However, considering that peroxisomal matrix proteins acquire at least part of their tertiary structure already in the cytosol, prior to the translocation step, it is clear that the DTM must contain a rather large pore to accommodate PEX5 plus its cargo ([52,53,54,55]; reviewed in ref. [56]). Interestingly, both PEX14 and PEX13 contain large intrinsically disordered regions, a property that may explain the flexibility that the DTM must possess in order to accommodate folded cargo proteins of so different sizes and shapes (reviewed in ref. [57]).

The interaction between cytosolic PEX5 and the DTM is a regulated event—only cargo loaded PEX5 molecules have access to the DTM ([58] and reviewed in ref. [57])—and occurs in two sequential steps: (1) docking, a reversible interaction and (2) insertion, an essentially irreversible event in the absence of ATP [18,59]. Importantly, PEX5 at the insertion stage displays a transmembrane topology, exposing a small N-terminal domain into the cytosol whereas most of its polypeptide chain faces the organelle matrix [60]. Similarly, a portion of the polypeptide chain of PEX7 also becomes transiently exposed to the organelle matrix during the PTS2 protein transport cycle [61].

Several interactions between PEX5 and DTM components have been characterized in vitro. By far the strongest one involves a small globular domain in the N-terminus of PEX14, which faces the organelle matrix, and a set of eight “Short Linear Motifs” (SLiMs; [62]), the so-called pentapeptide motifs, which are present in the N-terminal half of PEX5 ([63] and reviewed in ref. [57]). Each of these PEX5 SLiMs interacts with PEX14 quite strongly (K_d_ values from 7 to 157 nM) resulting in a high affinity/high avidity interaction between PEX5 and PEX14 at the DTM [63,64].

Insertion of cargo loaded PEX5 into the DTM culminates in the release of the cargo protein into the organelle matrix [17,18,61]. The available evidence suggests that no ATP hydrolysis is required also at this step and that release of the cargo is probably triggered by allosteric regulation of PEX5 by a DTM component (e.g., PEX14, PEX13, or in yeasts also PEX8) [16,18,65,66,67,68,69,70,71].

At the end of the cargo translocation step, the strong interactions between PEX5 and the DTM have to be disrupted. This is a complex process involving many proteins. Some of these are required to ubiquitinate DTM-embedded PEX5 at a cysteine residue near its N-terminus (Cys11 in the mammalian protein), a mandatory modification for the extraction event [72,73]. These proteins include defined ubiquitin-conjugating enzymes, which may be different in different organisms and one or several RING proteins of the DTM that function as ubiquitin-ligases in this reaction (reviewed in ref. [74]). Another set of proteins is required to extract monoubiquitinated PEX5 (Ub-PEX5) from the DTM. This set comprises PEX1, PEX6 and a poorly conserved tail-anchored membrane protein called PEX26 in mammals and many other organisms, PEX15 in yeasts/fungi and APEM9 in plants [75,76,77,78,79,80]. PEX1, PEX6 and this membrane protein form a protein complex at the peroxisomal membrane, the Receptor Export Module (REM) [81]. The available structural/functional data on the REM are discussed below.

## 3. The Receptor Export Module—the Initial Findings

The genes encoding REM components were identified in yeasts, mammalian cell lines, and plants using genetic approaches many years ago [41,75,79,82,83,84,85,86,87,88]. Subsequent homology searches revealed their conservation in many other organisms (reviewed in refs. [89,90]). Of relevance, mutations in two REM components, PEX1 and PEX6, have been shown to be the most frequent cause of peroxisomal biogenesis disorders accounting for 76% of all cases [91,92,93].

Primary structure analyses of PEX15/PEX26/APEM9 did not provide much insight into their function [75,79,94]. In contrast, PEX1 and PEX6 turned out to be members of the large family of ATPases Associated with diverse cellular Activities (AAA+), and the only proteins involved in peroxisomal matrix protein import possessing ATP binding/hydrolysis domains [95,96]. This finding, together with previous experiments showing that the overall process of peroxisomal protein import requires ATP hydrolysis [97,98,99,100,101] led to initial models proposing that PEX1 and PEX6 might participate at a pre-translocation step (e.g., in the disassembly of PEX5-cargo protein complex), or in the vectorial transport of proteins across the organelle membrane, or simply in the assembly of the DTM (reviewed in ref. [102]). However, subsequent experiments using in vitro import assays revealed that ATP hydrolysis in the peroxisomal protein import pathway is required only at a post-translocation stage, to extract PEX5 from the peroxisomal DTM [16]. In agreement with this, it was later reported that adding PEX1/PEX6-containing cytosolic fractions to organelles isolated from cells lacking PEX1/PEX6 restored the extraction process of PEX5 from those organelles [77,78]. It became then accepted that the function of PEX1/PEX6 is to extract PEX5 from the DTM, i.e., to reset the peroxisomal protein import machinery. We note that for many years PEX1/PEX6 were also believed to play a role in membrane vesicle fusion events, which presumably occur at early stages of peroxisome biogenesis [103,104]. However, a recent study found that cells lacking PEX1 or PEX6 actually contain peroxisomal “ghosts” which already possess membrane proteins; these “ghosts” are replenished with matrix proteins upon reintroduction of PEX1 or PEX6 [105]. In another work, it was shown that depleting PEX1 from yeast cells blocks import of matrix proteins without affecting biogenesis of the peroxisomal membrane [106]. Thus, a role of PEX1 and PEX6 in early steps of peroxisomal membrane biogenesis seems rather unlikely [105,106].

## 4. The Mechanism of the Receptor Export Module

**Figure 2:**
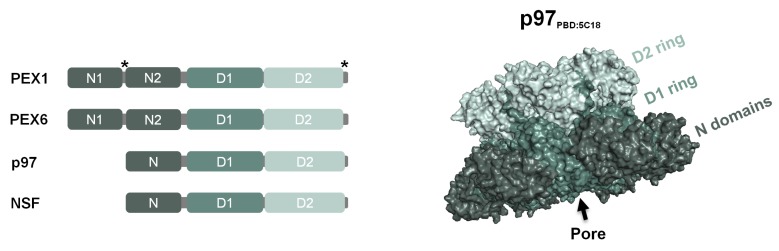
Domain architecture of the type II AAA+ proteins PEX1, PEX6, p97 and NSF. The two N-domains of PEX1 and PEX6 (N1 and N2) homologous to the N-domain of p97 and NSF (N-domain), and the two AAA+ domains (D1 and D2) are shown (left). Note that mammalian PEX1 is longer than the yeast protein, possessing a different spacing between its N-domains, it also has an extended C-terminal tail after the D2 domain (marked with asterisks). Atomic model of a p97 hexamer (right; PDB code 5C18, [138]) showing the common type II AAA+ structure consisting of one ring of D1 domains on top of another ring of D2 domains, and the six N-domains located on the side of the D1 ring. Note that this p97 structure was obtained in the presence of a non-hydrolysable ATP analogue and in the absence of substrate [138].

As stated above PEX1 and PEX6 are members of the AAA+ family, a large group of ATPases involved in a myriad of biological pathways such as DNA replication (e.g., DnaA), membrane fusion events (e.g., N-ethylmaleimide sensitive factor (NSF)), protein unfolding/disaggregation (e.g., p97/CDC48) and proteolysis (e.g., the 26S proteasome) [107,108,109,110]. AAA+-type ATPases use energy from ATP binding and hydrolysis to perform mechanical work which is used to unfold or remodel substrates [111]. The defining structural feature of these proteins is the AAA+ domain, a 200–250 amino acid residues ATP-binding domain comprising Walker A and Walker B motifs, and the second region of homology (SRH) domain that contains the sensor 1 and arginine finger motifs, key elements for the hydrolysis of ATP (reviewed in ref. [112]). The number of AAA+ domains in these proteins is variable but most have one or two such domains; these are referred to as type I or type II AAA+ proteins, respectively. Frequently, the AAA+ domain(s) is(are) flanked by additional domains which may be involved in interactions with adaptors and substrates or in regulating the activity of the AAA+ proteins (see refs. [113,114] for some recent examples; reviewed in refs. [115,116,117,118]) (see Figure 2).

Proteins with one or two AAA+ domains assemble into homo- or hetero-oligomers often yielding ring-shaped hexamers [108,112]. Such organization places the ATP-binding/hydrolysis site at the interface of two subunits, one of which provides the Walker A/B and sensor 1 residues and the other the arginine finger and creates a central pore that is frequently of major functional importance [112,119]. Indeed, many AAA+-type ATPases use specific residues in their pores, the so-called pore loops, to grasp substrates while others use the pore loops as paddles to translocate substrates through the pore, thus unfolding them ([120,121,122]; reviewed in refs. [96,112]). Paradigmatic examples of these two mechanisms are provided by NSF and p97/CDC48, two type II AAA+ proteins. NSF is believed to interact with its substrate, a SNARE complex, through its pore loops and to use ATP binding/hydrolysis to induce large movements in its N-domains which in turn are propagated to the substrate to disassemble it [123,124,125,126,127]. P97/CDC48 captures an extended/disordered domain of a substrate in its pore and then uses ATP binding/hydrolysis to thread the substrate through the pore [121,128,129,130]. Like NSF and p97/CDC48, PEX1 and PEX6 are also AAA+-type II ATPases. Besides the two AAA+ domains, both PEX1 and PEX6 possess also two N-terminal domains (N1 and N2), each displaying sequence similarities with the single N-domain present in p97/CDC48 and NSF (see Figure 2; [131] and reviewed in ref. [109]). Intriguingly, while the second AAA+ domain of both PEX1 and PEX6 (the D2 domain) displays all the canonical residues involved in ATP binding/hydrolysis, their first AAA+ domain (the D1 domain) is somewhat degenerated lacking many of the residues required for ATP hydrolysis [41,82,83,84,87,88,132,133,134,135,136,137].

Despite all the information provided by the primary structures of PEX1/PEX6, the actual mechanism used by the REM to extract monoubiquitinated PEX5 from the DTM remained a mystery for many years. A major breakthrough on this issue came in 2015 with the structural characterization of the yeast PEX1/PEX6 complex by negative-stain and cryo-electron microscopy [132,133,137]. That work revealed that the two proteins form a hetero-hexameric ring with alternating PEX1 and PEX6 subunits and a rather large pore at the center. Besides showing the typical arrangement of the AAA+ domains in type II ATPases—a ring of D1 domains on top of another ring made of D2 domains—the structures also revealed the position of the two PEX6 N-domains and the N2 domain of PEX1—they stand on the top/side of the D1 ring giving the complex a triangular shape [132,133,137]. More recently, the structure of the cytosolic domain of the tail-anchored PEX15 (amino acid residues 1-331) was also delineated [139]. The protein comprises a N-terminal disordered region of 42 amino acid residues, which mediates the interaction with PEX6 [76,139,140,141,142], a compact curved domain comprising 12 alpha-helices (residues 43–253) followed by a long intrinsically disordered region (residues 254–331) that connects the compact domain to the single transmembrane domain at the C-terminus of PEX15 [139]. It was also shown that three molecules of the soluble PEX15 cytosolic domain bind to the hexameric PEX1/PEX6 complex through the N1 and N2 domains of PEX6, and that the C-terminal intrinsically disorder region of the PEX15 fragment sits just above the central pore of the PEX1/PEX6 complex [139,143].

Another finding of crucial importance to understand the mechanism of the REM was made by Gardner et al. when studying the ATPase activity of the isolated yeast PEX1/PEX6 complex in vitro [133,139]. These authors noted that the complex displays a rather large basal ATPase activity which, however, is largely decreased in the presence of a recombinant protein comprising the cytosolic domain of PEX15 (amino acid residues 1–327). Initially, it was reasoned that besides being the membrane anchor, PEX15 might also be a regulator/inhibitor of PEX1/PEX6 thus avoiding futile ATP-consuming cycles by the ATPase [133]. However, subsequent work by the same group demonstrated that the in vitro “inhibitory” effect of the PEX15 fragment required intact pore loops at the D2 domains of PEX1/PEX6 [139]. This observation strongly suggested that the PEX15 fragment was actually acting as a substrate for PEX1/PEX6 in those assays. Indeed, those authors were able to show that the complete polypeptide chain of that PEX15 fragment is unfolded by the PEX1/PEX6 complex, a phenomenon that was not observed with shorter PEX15 fragments lacking most of the C-terminal intrinsically disordered region [139]. Apparently, placing the long free disordered C-terminus of the PEX15 fragment just above the PEX1/PEX6 pore results in its engagement with the pore loops of the AAA+ complex and in the complete threading of the PEX15 fragment. These important results established PEX1/PEX6 as a processive protein translocase, thus placing the REM in the mechanistic group of well-characterized AAA+ proteins such as p97/CDC48 and HSP104 [139,144].

Considering that PEX15 as well as its mammalian orthologue, PEX26, interact with PEX14 [139,141,142,145,146], which in turn interacts with PEX5 at the DTM [50,51,63,64,142,145,146,147,148], the results above might suggest that PEX15 is a physiologically relevant substrate for the PEX1/PEX6 complex and that remodeling of PEX15 by the REM somehow disrupts the interaction between PEX5 and the DTM. However, it must be noted that the C-terminus of the PEX15 fragment engaged by PEX1/PEX6 in those in vitro assays is actually linked to a transmembrane domain in the native/peroxisomal PEX15 protein. Thus, one would have to hypothesize that the PEX1/PEX6 complex engages a PEX15 loop in vivo, which remains to be demonstrated. Perhaps more importantly, it would be difficult to understand how unfolding the cytosolic domain of PEX15, or part of it, by PEX1/PEX6 might lead to the disruption of the PEX5-PEX14 interaction, most of which occurs on the other side of the peroxisome membrane [149]. Thus, as discussed by Gardner et al., the physiologically relevant substrate(s) of PEX1/PEX6 might well be other protein(s) [139].

A prime candidate substrate for the REM is of course DTM-embedded monoubiquitinated PEX5 itself, the only protein that is actually extracted from the DTM by the REM [16,59,77,78]. Indeed, using a mammalian cell-free in vitro system that recapitulates all the steps of the PEX5-mediated protein import pathway, we recently reported several observations that support this possibility [80]. First, it was shown that the ubiquitin moiety of DTM-embedded monoubiquitinated PEX5 interacts directly with both PEX1 and PEX6. Second, fusion proteins comprising PEX5 or its N-terminal half and GFP or mouse DHFR, respectively, were shown to enter the DTM and acquire a monoubiquitin; however, although export of these species can be initiated it does not terminate efficiently leading to the accumulation of partially extracted PEX5 proteins at the organelle surface [80,150]. In the case of the PEX5-DHFR fusion protein this effect was particularly noted when the assay contained methotrexate, a folate analog that stabilizes the structure of DHFR [151]. Thus, difficult to unfold moieties attached to the C-termini of PEX5 proteins interfere with the REM. Finally, cysteine residues present in the globular C-terminal half of PEX5 and located dozens/hundreds of amino acid residues away from the PEX5 domains that interact with DTM components, become particularly exposed to an alkylating reagent during (but not before nor after) the ATP-dependent export of Ub-PEX5 by the REM [80]. Altogether, these data suggest that Ub-PEX5 interacts with the REM and is completely unfolded during the export step.

## 5. Unsolved Mechanistic Aspects: Substrate Engagement and Regulation

**Figure 3:**
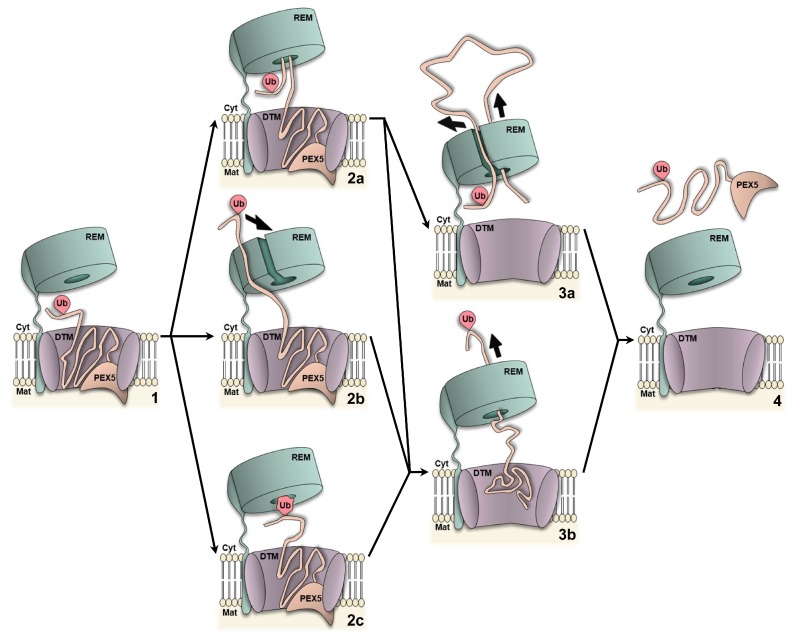
Possible mechanisms of PEX5 engagement and extraction by the REM. DTM-embedded Ub-PEX5 (**1**) may engage the REM in different ways. Given the intrinsically disordered nature of its N-terminal half, an extended loop of PEX5 could serve as the initial grabbing site for the AAA+ pore loops (**1→ 2a**; see [156] for a mechanism of this type). Alternatively, the AAA+ complex could assemble around PEX5 or undergo conformational changes to accommodate the substrate in its pore, as suggested for microtubule-severing enzymes [159]; this would place the Ub moiety already at the trans side of the AAA+ ring (**1→ 2b**). In a third scenario, local unfolding and threading of the Ub moiety itself by the AAA+ proteins could also be used to extract Ub-PEX5 (**1→ 2c**), akin to a mechanism recently proposed for the CDC48 ATPase complex [129]. Threading of Ub-PEX5 by the AAA+ proteins could either be partial (paths **2a→ 3a** and **2b→ 3b**; the ubiquitin moiety is not threaded) or complete (paths **2a→ 3b** and **2c→ 3b**; the ubiquitin is threaded). 1—Recognition; 2—Engagement; 3—Translocation; 4—Release.

Many AAA+-type ATPases, such as those of the proteasome 19S regulatory particle or the microtubule severing katanin, engage substrates by capturing their extended N- or C-termini into their pores [152,153,154,155]. This, however, probably does not apply to the REM because (1) a truncated human PEX5 protein lacking its first nine amino acids (a deletion that does not interfere with monoubiquitination of PEX5 at residue 11) is still efficiently extracted by the REM and (2) several C-terminally truncated PEX5 proteins (one of which comprising solely amino acid residues 1–125 of PEX5) or PEX5 fusion proteins containing folded domains at the C-termini are, likewise, monoubiquitinated at the DTM and engaged by the REM [55,59,80,150]. These data nevertheless suggest that amino acid residues 10–125 of PEX5 plus the covalently attached ubiquitin moiety are sufficient for engagement with the REM. Exactly how this occurs awaits further experimentation but two obvious scenarios are the following (see also Figure 3): (1) the REM engages Ub-PEX5 at a protein loop in the 10–125 region of PEX5 (see [156] for a similar mechanism) or (2) PEX1/PEX6 oligomerize around a PEX5 domain located after the ubiquitination site. A third, less evident mechanism may also be envisaged when we take into consideration very recent data showing that CDC48 engages a polyubiquitinated substrate by unfolding one of its ubiquitin moieties [129]. In such a scenario, the REM would first unfold the ubiquitin moiety of Ub-PEX5 at the entrance of the PEX1/PEX6 pore, and then would engage the ubiquitin N-terminus (see Figure 3). Although this possibility might seem unlikely due to the fact that the D1 domains of PEX1/PEX6 lack many of the residues required for ATP hydrolysis [82,83,84,86,88,132,133,137], we note that inactivating mutations in the walker B domain or arginine finger of the D1 domain in all six subunits of the bacterial ClpB protein do not completely abolish its disaggregase activity [113]. Clearly a type II ATPase containing only fully functional D2 domains can still do the job (see also refs. [157,158]).

As stated above, the isolated yeast PEX1/PEX6 complex displays a large basal ATPase activity in vitro in the absence of any substrate [133,139,160]. Naturally, futile hydrolysis of ATP should not occur in vivo thus raising the question of how the ATPase activity of the REM is regulated. One possibility is that a still unidentified protein (or protein domain) interacts with the PEX1/PEX6 complex repressing its ATPases activity and disengaging from the ATPase in the presence of the substrate. Such a regulatory protein might be any component of the DTM or even PEX15/PEX26 itself—the properties of this PEX1/PEX6-interacting protein in its native environment, i.e., embedded in the peroxisomal membrane, may be different from those presented by its soluble cytosolic domain in the in vitro assays reported above [139]. Another possibility comes from models proposed for other members of the AAA+ family such as the microtubule severing enzymes, katanin and spastin [159,161] or the GspE protein from bacterial type II secretion systems [162]. It has been proposed that these AAA+ proteins undergo oligomerization, yielding an active ATPase, only under specific conditions. In the case of the microtubule severing enzymes, oligomerization seems to occur only after the corresponding subunits bind microtubules [161,163]. In the case of GspE, oligomerization might also be substrate-induced but in an indirect manner, probably via a substrate-sensing subunit of the secretion system [162]. Clearly, additional work is needed to understand these important mechanistic aspects of the REM. 

## 6. The Energetic Cost of Protein Translocation across the Peroxisomal Membrane

As discussed in the previous sections, the peroxisomal protein import machinery relies on the strong protein–protein interactions that are established between PEX5 and the DTM as the driving force for protein translocation, and uses ATP hydrolysis only to recycle PEX5 back into the cytosol [16,17,18]. One implication of such a mechanism is that the energetic cost of translocating small or large proteins across the peroxisomal membrane is the same. What is this cost exactly? The number of ATPs consumed during dislocation of Ub-PEX5 from the DTM depends on the number of amino acid residues of Ub-PEX5 that have to be threaded through the PEX1/PEX6 pore, and on the step size of the PEX1/PEX6 complex during threading. As discussed in detail above, the available data suggest that engagement of Ub-PEX5 by the REM involves the ubiquitin moiety itself plus amino acid residues 10–125 of PEX5 and that threading continues to the very C-terminus of the PEX5 protein [55,80,150]. Thus, practically all PEX5 residues (approximately 600) are threaded through the PEX1/PEX6 pore. The translocation step size of PEX1/PEX6 is still unknown. There are indirect estimates suggesting an energetic cost of 1 ATP per seven amino acid residues threaded [139], similarly to other AAA+ proteins (e.g., ClpA [164]). However, other type II translocases, such as p97/CDC48 and bacterial ClpB work with step sizes of just two amino acid residues, with ATP hydrolysis occurring in both D1 and D2 domains [113,129,130]. Thus, a conservative estimate for the PEX1/PEX6 complex, taking also into consideration that its D1 ring probably does not hydrolyze ATP, would be one ATP per 2–7 amino acid residues of substrate translocated through the pore. This means that the complete dislocation of Ub-PEX5 from the DTM requires the hydrolysis of some 85–300 ATPs. These are relatively small numbers when compared, for instance, to the energy required for translocating a protein (prOmpA) across the bacterial plasma membrane through the Sec pathway (700–5000 ATPs) [165] or to transport a protein (OE17) using the chloroplast Tat pathway (690,000 kJ/mol, equivalent to 11500 ATPs) [166]. Apparently, the peroxisomal protein import machinery is rather economical.

## 7. Conclusions

Over the last 5 years, we have learned more on the REM structure and function than in the two previous decades. We now know that the PEX1/PEX6 complex is a p97/CDC48-like ATPase that threads monoubiquitinated PEX5 through its central pore. Naturally, there are still many questions waiting for an answer. As discussed above we still have no idea of how Ub-PEX5 is engaged by the PEX1/PEX6 complex, and how this ATPase is kept in an inactive state in the absence of substrates. Also, it is still unknown whether the PEX1/PEX6 complex has other substrates besides PEX5 and all the yeast/fungi PEX5-like proteins. Hopefully, we will not need two more decades to answer these questions.

## Abbreviations

AAA+	ATPases Associated with diverse cellular Activities
Cys11	Cysteine 11
Cyt	Cytosol
D1	AAA+ domain 1
D2	AAA+ domain 2
DHFR	Dihydrofolate reductase
DTM	Docking/translocation module
GFP	Green fluorescent protein
K_d_	Dissociation constant
Mat	Matrix
N1	N-terminal domain 1
N2	N-terminal domain 2
NSF	N-ethylmaleimide sensitive factor
PTS	Peroxisomal targeting signal
REM	Receptor export module
RING	Really interesting new gene
SLiMs	Short linear motifs
SRH	Second region of homology
Tat	Twin-arginine translocation
Ub	Ubiquitin
Ub-PEX5	Monoubiquitinated PEX5
